# Assessment of Drug Information Service in Public and Private Sector Tertiary Care Hospitals in the Eastern Province of Saudi Arabia

**DOI:** 10.3390/pharmacy5030037

**Published:** 2017-07-04

**Authors:** Sawsan Abdullah Alamri, Raniah Ali Al Jaizani, Atta Abbas Naqvi, Mastour Safer Al Ghamdi

**Affiliations:** 1 College of Clinical Pharmacy, Imam Abdulrahman Bin Faisal University (University of Dammam), Dammam 31441, Saudi Arabia; sawsan.ama94@gmail.com; 2 Department of Pharmacy Practice, College of Clinical Pharmacy, Imam Abdulrahman Bin Faisal University (University of Dammam), Dammam 31441, Saudi Arabia; raaljaizani@uod.edu.sa (R.A.A.J.); bg33bd@student.sunderland.ac.uk (A.A.N.)

**Keywords:** drug information service, pharmacists, Saudi Arabia

## Abstract

Drug information service is a dedicated and specialized service provided by pharmacists to enhance knowledge of medicines use, promote rational prescribing among prescribers, and reduce medication errors. Saudi Arabia has a National Drug and Poison Information Center (NDPIC) responsible for answering drug queries. There is a lack of literature that reports the current scenario of drug information services in the country, especially the Eastern Province. This study reported the current status of drug information services being provided among tertiary care hospitals of the Eastern Province of Saudi Arabia. All hospitals provided drug information services. The qualification of personnel was mostly bachelor’s level (46.2%) and without proper training (54.8%). The most common queries received in a day were related to drug alternatives, dosage, and administration, as well as the availability of drugs. Physicians were the main users of the service. The most common health resources employed for the service was Lexi-Comp (76.9%) and Micromedex (69.2%). The use of Saudi National Formulary was not reported by any hospital, which highlights a potential research gap to address i.e., to investigate the lack of use of SNF by practitioners.

## 1. Introduction

Drug information service is a specialized service provided by pharmacists to enhance drug knowledge, empower rational prescribing, and reduce medication errors [1]. This service is provided in response to the queries sought by allied health professionals in addressing medication-related problems pertaining to pharmacotherapy and medicine management issues of patients [2,3]. One of the most important aspects of drug information is to be unbiased in its contents [4]. Thus, the unbiased nature of information is of paramount importance to enhance patient outcomes and reduce adverse drug reactions (ADRs) [5].

Saudi Arabia has a National Drug and Poison Information Center (NDPIC) responsible for answering drug queries. The center has a total of 31 hospitals with established drug information centers. It is distributed in the following manner: the Central region has 11 established drug information centers, while there are nine in the Eastern region, three in the South, five centers in the West and two drug information centers in the Northwestern region of the country [6]. The country’s health authority has a policy on the minimum standards required for drug information services that includes organizational and clinical objectives [7]. However, there is a dearth of research literature reported in Saudi Arabia on the quality parameters of this service, especially in the Eastern region with regard to the work operations of drug information services provided in tertiary care hospitals.

## 2. Methods

This descriptive study, which was five months in duration (January 2017 to May 2017), was conducted in five cities including Dammam, Dhahran, Al Hufuf, Khobar, and Qateef, located in the Eastern Province of Saudi Arabia. The study incorporated tertiary care hospitals across five cities of the Eastern Province of Saudi Arabia i.e., seven hospitals in Dammam (53.8%), two hospitals (15.4%) each in Dhahran and Al Hufuf, and one hospital each (7.7%) in Khobar and the city of Qateef. This amounted to a total of 13 healthcare facilities to assess drug information services. The data source or target population was the pharmacy department of the hospital, particularly the head of the pharmacy department or pharmacy manager. The study used a structured, closed-ended survey questionnaire to assess the service parameters, which was exclusively developed by reviewing the literature. The survey questionnaire contained a total of 10 questions. It included questions related to the demographic information of the drug information personnel, quality assessment information such as mode of delivery, frequency of information sought, type of drug information sought, and the persons seeking such information, lag time, etc., as well as the use of resources to obtain such information. Prior permission was sought from the hospitals before the commencement of the study. The survey was conducted among the hospital pharmacy managers by dropping off the questionnaire and collecting it again at their time of choosing. The study was noninvasive in nature and the participants were briefed about the objectives before completing the questionnaire. The study was exempted from review, however, a letter addressed to the concerned hospital was produced on request.

## 3. Results

Most of the hospitals were in the public sector (*N* = 10, 76.9%) while some were in the private sector (*N* = 3, 23.1%). All hospitals (*N* = 13, 100%) confirmed that they provide drug information services. Moreover, almost half of the hospitals (*N* = 7, 53.8%) had a dedicated drug information office followed by a third proportion (*N* = 4, 30.8%) that provided the service through a clinical pharmacy department. One hospital utilized an on-duty pharmacist and another hospital reported that anyone, whether pharmacist, pharmacy manager, or a technician present on the telephone line in the pharmacy department provided the service.

Furthermore, the qualification of the personnel was mostly bachelor’s level i.e., Bachelors in Pharmacy (B.Pharm) (*N* = 6, 46.2%) and Doctor of Pharmacy (Pharm.D) (*N* = 2, 15.4%), followed by few hospitals (*N* = 4, 30.8%) which declined to mention the qualification of the staff responsible for the service. One hospital had a pharmacist with Master’s qualification. Additionally, almost half of the hospitals (*N* = 6, 46.2%) had previously trained staff for the said purpose followed by a third (*N* = 4, 30.8%) that did not mention the details, and almost a quarter (*N* = 3, 23.1%) that did not have such training. The mode of inquiry was mainly direct access and via telephone (*N* = 5, 38.5%), followed by emails (*N* = 4, 30.8%) and direct access alone (*N* = 2, 15.4%). One hospital provided drug information service by telephone only and another by all modes.

Additionally, information relating to the quality assurance (QA) of the service was also sought from the hospitals. In this context, we found out that the majority of the hospitals provided immediate response to the drug information queries received, but only half of them kept a record for quality assurance purposes. The response was mostly verbal. Moreover, only two hospitals assimilated that information to produce newsletters. This hospital focused on continuous education programs for the patients as well as healthcare professionals. The data pertaining to the quality of service is tabulated in Table 1.

With regard to the resources used for answering queries, we found that most of the hospitals used Lexi-Comp’s Drug Information Handbook (*N* = 10, 76.9%) and Micromedex (*N* = 9, 69.2%). The data is presented in Table 2.

Furthermore, the frequency of different types of drug information queries received at the pharmacy was also sought from the hospitals. The most common queries were related to seeking drug alternatives, administration and dosage, compatibility and safety issues, contraindications and availability of drugs in the pharmacy. Queries regarding adverse drug reactions (ADRs), pregnancy and lactation issues, as well as drug interactions were sought occasionally. Information regarding the toxicological parameters of drugs was rarely sought. The summary of these results is tabulated in Table 3.

The frequency of queries received per day from allied healthcare professionals and patients was also documented. Most commonly, the queries were received from physicians and pharmacists followed by nurses. The drug information service sometimes received queries from patients and interns. The summary of these results is presented in Table 4.

## 4. Discussion

Our study revealed that drug information service is now provided in all major tertiary care hospitals across five cities in the Eastern Province. Though all hospitals provide the service, most of the pharmacists providing the information had bachelor’s qualification and only half of them had specialized training. This is not in accordance with the standards normally assumed for the service. While all pharmacists can provide drug information to consumers and healthcare professionals to some extent, the quality of the service is believed to improve if the pharmacist has had formal training or supervision of a drug information specialist [8]. This will offer greater support in combating medication errors, as expert pharmacists will be more poised and experienced in countering such problems. Hence, incorporating qualified and trained pharmacists may be more beneficial for achieving positive patient outcomes. The study also reported that the most common health resources employed for the service was Lexi-Comp and Micromedex. A similar study conducted among pharmacy students and faculty at a university in the USA also reported the high preference for Lexi-Comp for seeking information [9]. Secondly, the increased use of Micromedex was also reported from hospitals across the globe [1,10].

Our study also found that most of the queries were received from physicians. Studies conducted in other parts of the world also observed physicians being the most common enquirers of drug information [1,11]. The information related to the type of query was also sought from drug information personnel. We found that most the queries received in a day were related to drug alternatives, dosage and administration, as well as the availability of drugs. This finding was unusual, as it reported a high frequency of queries related to dosage and administration which was previously reported in studies conducted in different locations. However, it also observed that queries related to ADRs were reported rarely. This observation is contradictory to previous findings, as studies conducted in a hospital in India reported that a quarter of the total queries received were of such a nature [1]. Furthermore, the study observed that the other most common queries were the availability of the drug in the pharmacy and the drug alternatives. This highlights the growing use of online resources by physicians to seek ADRs and toxicology profiles of drugs to prescribe. Hence, physicians only sought information pertaining to the logistical constraints.

Since the common resources used did not contain information about the local availability and alternatives available in Saudi Arabia, perhaps a better solution to this problem may be the use of the Saudi National Formulary (SNF), as it can serve as a better alternative to other resources in such situations. The Saudi National Formulary contains drug information tailored for use in the country with related information [12]. However, the formulary needs to be updated more frequently to incorporate the latest information and must be freely available to all practicing healthcare professionals in Saudi healthcare settings. Further investigation is needed to find out the reasons that led to the decreased or lack of use of the SNF among healthcare practitioners in Saudi Arabia. The service should also field calls pertaining to the questions related to non-emergent cases and must promote direct interactions with the patients. This practice may increase the patient-pharmacist interaction, invest patient trust in pharmacists, and have the potential to achieve positive patient outcomes. One of the minimum standards pertaining to drug information services in Saudi Arabia is to monitor and report medication errors [7]. This standard has been mentioned in the official directives, however, it still needs to be put in practice.

Overall, it is believed that the drug information service may be improved by an upgrade of personnel i.e., pharmacists with higher qualification, training in drug information service, and appropriate experience should be hired, followed by updating the Saudi National Formulary (SNF) and establishing a mechanism for medication error identification and reporting.

## 5. Conclusions

Drug information service was provided in all major healthcare settings. Pharmacists working as a drug information specialist mostly held a bachelor’s degree in pharmacy, with most of them not having any specialized training in the domain. This issue needs to be addressed for the future as it may be considered as a shortcoming and may require upgradation. Physicians benefitted the most from the drug information service, however, their queries were mostly related to the logistical constraints of drug availability and alternatives. Overall, the service is adequate. The use of the Saudi National Formulary was not reported by any hospital, which highlights a potential research gap to address i.e., to investigate the decreased use of the SNF by practitioners. This study will help understand the potential areas for improvement of the service and the ways in which drug information service in the country can contribute to positive patient outcomes.

## Figures and Tables

**Table 1:** Details of drug information service.

Information (*N* = 13)	Sample (*N*)	Percentage (%)
**Keeping Record of Received Queries**		
Yes	7	53.8
No	6	46.2
**Average Response Time**		
Immediately	10	76.9
Within 24 h	3	23.1
**Most Common Mode of Response**		
Verbal	4	30.8
Verbal and written	4	30.8
Verbal and written and mail	2	15.3
Mail	1	7.7
Verbal and printed literature	1	7.7
Printed literature	1	7.7
**Participate in Patient Education Programs**		
Yes	12	92.3
No	1	7.7
**Report Adverse Drug Reactions (ADRs)**		
Yes	8	61.5
No	5	38.5
**Provide Educational Program for Healthcare Professionals**		
Yes	9	69.2
No	4	30.8
**Produce Newsletter**		
Yes	2	15.4
No	11	84.6

**Table 2:** Resources used in hospitals.

	Resources Used (Online Included)	Type of Hospitals (*N* = 13)		
		Public Yes (No)	Private Yes (No)	Total Yes (%)/No (%)
1	Lexi-Comp’s Drug Information Handbook	7 (3)	3 (0)	10 (76.9)/3 (23.1)
2	Micromedex	7 (3)	2 (1)	9 (69.2)/4 (30.8)
3	Iowa Drug Information Service	1 (9)	0 (3)	1 (7.7)/12 (92.3)
4	Uptodate	6 (4)	1 (2)	7 (53.8)/6 (46.2)
5	Meyler’s Side Effects on Drugs	1 (9)	1 (2)	2 (15.4)/11 (84.6)
6	Martindale’s Extra Pharmacopoeia	2 (8)	1 (2)	3 (23.1)/10 (76.9)
7	American Hospital Formulary Service (AHFS) Drug Information	1 (9)	0 (3)	1 (7.7)/12 (92.3)
8	Pharmacotherapy: A Pathophysiologic Approach	3 (7)	1 (2)	4 (30.8)/9 (69.2)
9	Applied Therapeutics: The Clinical Use of Drugs	4 (6)	2 (1)	6 (46.2)/7 (53.8)
10	Pediatric Dosage Handbook	3 (7)	1 (2)	4 (30.8)/9 (69.2)
11	Drugs in Pregnancy and Lactation	4 (6)	3 (0)	7 (53.8)/6 (46.2)
12	British National Formulary (BNF)	5 (5)	2 (1)	7 (53.8)/6 (46.2)
13	Australian Medicine Handbook	1 (9)	0 (3)	1 (7.7)/12 (92.3)

**Table 3:** Frequency of different queries sought from drug information services.

Nature of Query	Hospital	Frequency of Drug Queries			
		Never	Rarely	Sometimes	Commonly
**Adverse Drug Reactions**	Government	1	4	4	1
	Private	1	0	1	1
	Total	2	4	5	2
**Drug Alternatives**	Government	0	0	2	8
	Private	0	0	1	2
	Total	0	0	3	10
**Administration and Dosage**	Government	0	0	2	8
	Private	0	0	0	3
	Total	0	0	2	11
**Drug Interactions**	Government	0	3	3	4
	Private	0	0	2	1
	Total	0	3	5	5
**Drug Compatibility and Stability**	Government	0	1	4	5
	Private	0	2	0	1
	Total	0	3	4	6
**Drug Safety and Contraindications**	Government	2	2	3	3
	Private	0	1	0	2
	Total	2	3	3	5
**Availability of Drug in Pharmacy**	Government	0	0	1	9
	Private	0	0	2	1
	Total	0	0	3	10
**Drug Pharmacokinetics**	Government	5	2	2	1
	Private	2	1	0	0
	Total	7	3	2	1
**Drug Toxicity**	Government	1	4	4	1
	Private	0	1	1	1
	Total	1	5	5	2
**Pregnancy and Lactation**	Government	0	4	4	2
	Private	0	0	1	2
	Total	0	4	5	4

**Table 4:** Frequency of drug queries received from different persons.

Nature of Query	Hospital	Frequency of Drug Queries			
		Never	Rarely	Sometimes	Commonly
**Physicians**	Government	0	0	1	9
	Private	0	0	0	3
	Total	0	0	1	12
**Nurses**	Government	0	1	2	7
	Private	1	0	2	0
	Total	1	1	4	7
**Pharmacists**	Government	0	0	1	9
	Private	0	1	0	2
	Total	0	1	1	11
**Patients**	Government	1	1	6	2
	Private	0	0	1	2
	Total	1	1	7	4
**Interns**	Government	1	2	5	2
	Private	1	2	0	0
	Total	2	4	5	2
